# Longitudinal and Stability-Aware Analysis Reveals Treatment-Specific MicroRNA Response Signatures Following Immune-Reconstitution and B-Cell-Targeted Therapies in Multiple Sclerosis

**DOI:** 10.3390/ijms27114935

**Published:** 2026-05-29

**Authors:** Nasar Ata, Joshua S. Mytych, Mirela Cerghet, Ramandeep Rattan, Sumit Govil, Shailendra Giri, Yang Mao-Draayer

**Affiliations:** 1 Department of Neurology, Henry Ford Health, Detroit, MI 48202, USA; sata1@hfhs.org (N.A.); mcerghe1@hfhs.org (M.C.); 2 School of Life and Basic Science, Jaipur National University, Jaipur 302017, India; sumitgovil@jnujaipur.ac.in; 3 Oklahoma Medical Research Foundation, Oklahoma City, OK 73104, USA; josh-mytych@omrf.org; 4 Women’s Health Services, Henry Ford Health, Detroit, MI 48202, USA; rrattan1@hfhs.org

**Keywords:** multiple sclerosis, microRNA, cladribine, ocrelizumab longitudinal analysis, disease-modifying therapy, peripheral blood mononuclear cells (PBMCs)

## Abstract

Disease-modifying therapies (DMT)s) for relapsing-remitting multiple sclerosis (RRMS) act through distinct immunological mechanisms, yet the within-patient molecular response programs associated with these therapies remain incompletely defined. Here, we reanalyzed publicly available peripheral blood mononuclear cell (PBMC) miRNA microarray data (GSE230064) using a longitudinal, robustness-focused framework to compare therapy-associated miRNA response patterns following cladribine versus ocrelizumab treatment. Baseline (t0) and 6-month post-treatment (t1) samples were paired within individuals and technical replicates consolidated prior to analysis, yielding a final paired cohort of four cladribine-treated and six ocrelizumab-treated patients. Within each treatment arm, we quantified per-patient Δ-miRNA (t1 − t0) values and prioritized therapy-associated response features using a multi-evidence framework integrating effect direction, magnitude, directional consistency across individuals, and leave-one-out sensitivity. Cladribine treatment was associated with a highly coordinated, directionally concordant upregulation of five miRNAs including hsa-miR-27a-3p, hsa-miR-27b-3p, hsa-miR-503-5p, hsa-miR-148a-3p, and hsa-miR-26a-5p, all exhibiting 100% directional stability across patients and mean Δ-expression values ranging from +0.77 to +1.38. These miRNAs target pathways relevant to MS pathophysiology, including Th17/Treg balance, Wnt-β-catenin signaling, macrophage polarization, and epigenetic immune regulation. In contrast, ocrelizumab elicited a more selective response pattern, with five miRNAs including hsa-miR-100-5p, hsa-miR-410-3p, hsa-miR-432-5p, hsa-miR-296-5p, and hsa-miR-485-3p showing moderate directional stability (83%) and greater inter-individual heterogeneity, consistent with the more targeted mechanism of CD20+ B-cell depletion. Notably, the two treatment-associated signatures were non-overlapping, with hsa-miR-27b-3p representing the only miRNA shared with prior cross-sectional analyses of this dataset. The identified ocrelizumab-associated miRNAs implicate pathways including mTOR/IGF1R signaling, NF-κB regulation, RNA editing, and mitochondrial biogenesis, several of which are dysregulated in progressive MS. Together, these findings demonstrate that cladribine and ocrelizumab induce distinct, treatment-specific miRNA response architectures that reflect their divergent immunological mechanisms. This work establishes a stability-aware analytic template for extracting reproducible longitudinal miRNA signals from small paired RRMS cohorts and provides a ranked set of biologically plausible candidate miRNAs for prospective validation and mechanistic investigation.

## 1. Introduction

Multiple sclerosis [1] is a chronic immune-mediated demyelinating disorder [2] of the central nervous system. The clinical presentation is markedly heterogeneous: relapsing–remitting MS (RRMS) is characterized by recurrent episodes of neurological dysfunction and subsequent remission, secondary progressive MS (SPMS) is associated with a gradual deterioration in neurological function, and primary progressive MS (PPMS) presents with a gradual onset of progressive disability. RRMS is the most common form of the disease, with nearly 85% of patients presenting with this type at initial diagnosis [3] The variety in illness progression and treatment response necessitates that doctors primarily depend on clinical evaluation and MRI to detect disease activity; there is a persistent demand for reliable, non-invasive biomarkers that can reflect individual differences [4]. Herein, we utilize a publicly available RRMS patient cohort to identify differentially expressed microRNA (miRNA) biomarkers following disease-modifying therapy.

MiRNAs are about 22-nucleotide non-coding RNAs that modulate gene expression by facilitating the degradation or translational repression of target mRNAs [5]. Their diminutive size and encapsulation inside protein complexes or extracellular vesicles provide exceptional stability in serum, plasma, saliva, and cerebrospinal fluid [6]. Dysregulated miRNA networks influence the etiology of multiple sclerosis by adjusting the equilibrium between pro-inflammatory and regulatory T-cell subsets, modifying cytokine production, undermining blood–brain barrier integrity, and affecting oligodendrocyte development and remyelination [7]. Particular miRNAs, including miR-146a and miR-155, are activated by inflammatory stimuli and Toll-like receptor ligands, diminishing NF-κB signaling by targeting suppressors of cytokine signaling (SOCS1) and TNF receptor-associated factor 6 [8]. Due to their stability, ease of detection, and reflection of immunological and neurological processes, circulating miRNAs serve as appealing minimally invasive biomarkers for multiple sclerosis [5,9].

Increasing data substantiates the efficacy of miRNAs as indicators of disease activity, progression, and therapy response. Serum levels of miR-92a-3p and miR-486-5p exhibit an inverse correlation with MRI-defined lesion load, whereas elevated levels of miR-24-3p and miR-128-3p are linked to disability progression and relapse rate [10], respectively. Decreased blood concentrations of miR-223, miR-23a, and miR-15b distinguish primary progressive multiple sclerosis from healthy controls, but cerebrospinal fluid miR-142-5p expression escalates with clinical advancement [5]. Increased levels of “inflamma-miRs” such as miR-125a-5p, miR-34a, and miR-146a indicate active inflammation [5]. Treatment studies indicate that enhanced CSF miR-142-3p forecasts an unfavorable response to dimethyl fumarate [11], whereas natalizumab influences miR-126-3p [12], and increased serum miR-548a-3p distinguishes responders to fingolimod [13]. These data indicate that miRNAs are not only passive entities but active contributors to MS immunopathology and may function as molecular biomarkers that reflect disease heterogeneity.

MS treatments are classified in many ways and typically take a tiered approach; one of the most common is by efficacy. Low-efficacy drugs have higher safety but lower treatment effect (e.g., cladribine), while high-efficacy drugs yield a lower safety profile but show greater improvement in MS patient disability progression (e.g., ocrelizumab) [14]. Cladribine and ocrelizumab utilize different immunological pathways among disease-modifying medicines. Cladribine is a synthetic counterpart of deoxyadenosine; within lymphocytes, elevated intracellular levels enhance the activity of deoxycytidine kinase (DCK). DCK phosphorylates cladribine into its active form, which incorporates into DNA and interferes with DNA synthesis and repair, resulting in the death of B- and T-lymphocytes. Due to the elevated DCK activity in lymphocytes, cladribine selectively depletes memory B and T cells while preserving the majority of innate immune cells [15], resulting in a prolonged immunological reconstitution. Ocrelizumab is a humanized IgG1 monoclonal antibody that targets the CD20 antigen on late pre-B, naïve, and memory B cells, inducing antibody-dependent cellular cytotoxicity (ADCC), complement-dependent cytotoxicity, and death. CD20 is not present on plasma cells and hematopoietic stem cells; hence, ocrelizumab depletes circulating B cells while preserving antibody-producing plasma cells, facilitating repopulation from bone marrow progenitors [16]. In contrast to rituximab, ocrelizumab is more dependent on antibody-dependent cellular cytotoxicity (ADCC) and less on complement activation. The molecular distinctions indicate that cladribine may facilitate lasting immunological reconstitution, while ocrelizumab leads to persistent albeit reversible B-cell suppression.

Most current miRNA research studies in multiple sclerosis utilize cross-sectional approaches, which fail to capture intra-individual dynamics. To mitigate this restriction, our work utilizes publicly accessible miRNA data from peripheral blood mononuclear cells [17] and employs a longitudinal within-subject methodology. We aim to identify robust miRNA signatures linked to cladribine-mediated immune reconstitution versus ocrelizumab-mediated B-cell depletion by calculating patient-specific Δ-miRNA profiles (post-treatment minus baseline) and assessing effect size, directional consistency, and leave-one-out stability.

The initial study by [17] was largely concentrated on cross-sectional comparisons of miRNA expression among treatment groups, whereas the current study re-evaluates their RRMS patient dataset utilizing a longitudinal within-subject approach. By calculating patient-specific Δ-miRNA profiles (t1 − t0) and using directional consistency and leave-one-out stability measures, we uncover treatment-related miRNA signatures that are resilient to small cohort sizes and individual heterogeneity.

## 2. Results

**Figure 1:**
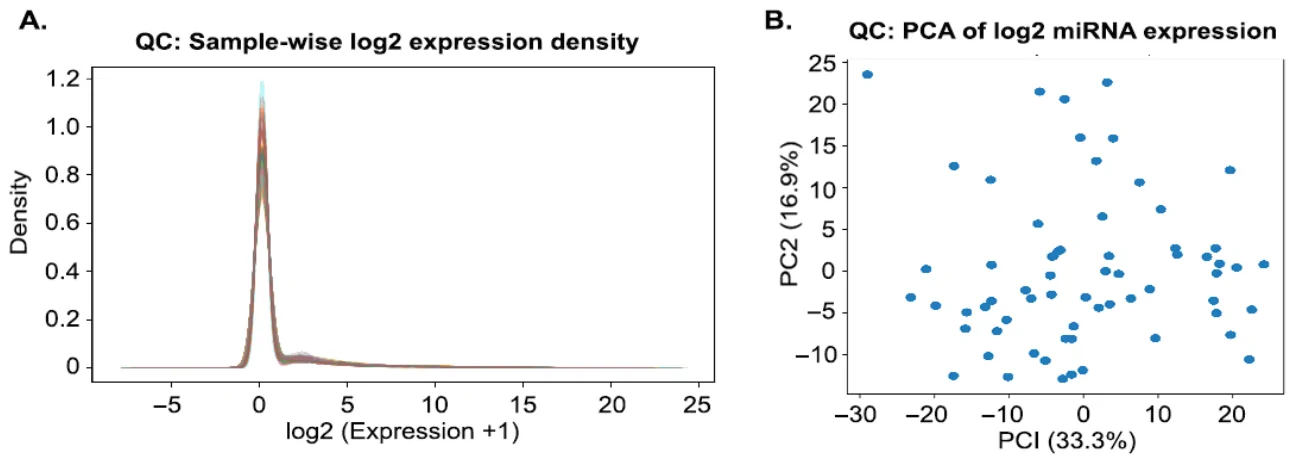
(**A**) Sample-wise density distributions of log2-transformed miRNA expression values across all samples, showing highly comparable global expression profiles. (**B**) Unsupervised principal component analysis (PCA) of log2-transformed miRNA expression values, demonstrating absence of extreme outliers or dominant technical effects.

Several additional quality control (QC) investigations were carried out to assess the overall quality and global structure of the miRNA expression data. All samples showed substantially overlapping distributions in sample-wise density plots of log2-transformed expression levels [18], suggesting that there was no significant technical bias and that global expression patterns were consistent (Figure 1A). The dataset’s general comparability was supported by the fact that no sample showed an aberrant density pattern or a significantly altered distribution.

The log2-transformed expression matrix was subjected to unsupervised principal component analysis (PCA), as previously demonstrated [19], which showed a continuous distribution of samples along the first two principal components. PC1 and PC2 accounted for a substantial proportion of the total variance and was without evidence of dominant technical effects [20] (Figure 1B). Crucially, neither a dominating technical axis nor any individual samples showed up as severe outliers, as previously seen [21], indicating that batch effects or technical abnormalities were not the primary causes of global variation. The consistency of median expression levels and interquartile ranges across samples was further supported by the overlapping distributional characteristics observed across samples.

### 2.1. Patient-Level Pairing and Longitudinal Sample Structure

Patient-level pairing identified a subset of treated individuals with complete longitudinal sampling suitable for within-subject analysis. From the full treated cohort, only patients with complete paired baseline and post-treatment samples were retained for longitudinal Δ-miRNA analyses, resulting in four cladribine-treated and six ocrelizumab-treated individuals. Six patients in the group treated with ocrelizumab satisfied the same requirements, whereas four patients in the group treated with cladribine had full baseline and post-treatment sample pairs. At least one technical replication was present for each timepoint in all paired patients, and few patients had more than one technical measurement at baseline and follow-up.

The number of patients with complete pairs and the existence of technical replicates at each timepoint are highlighted in Table 1, which provides an overview of the paired sample structure.

### 2.2. Δ-miRNA Profiles Following Cladribine and Ocrelizumab Treatment

**Figure 2:**
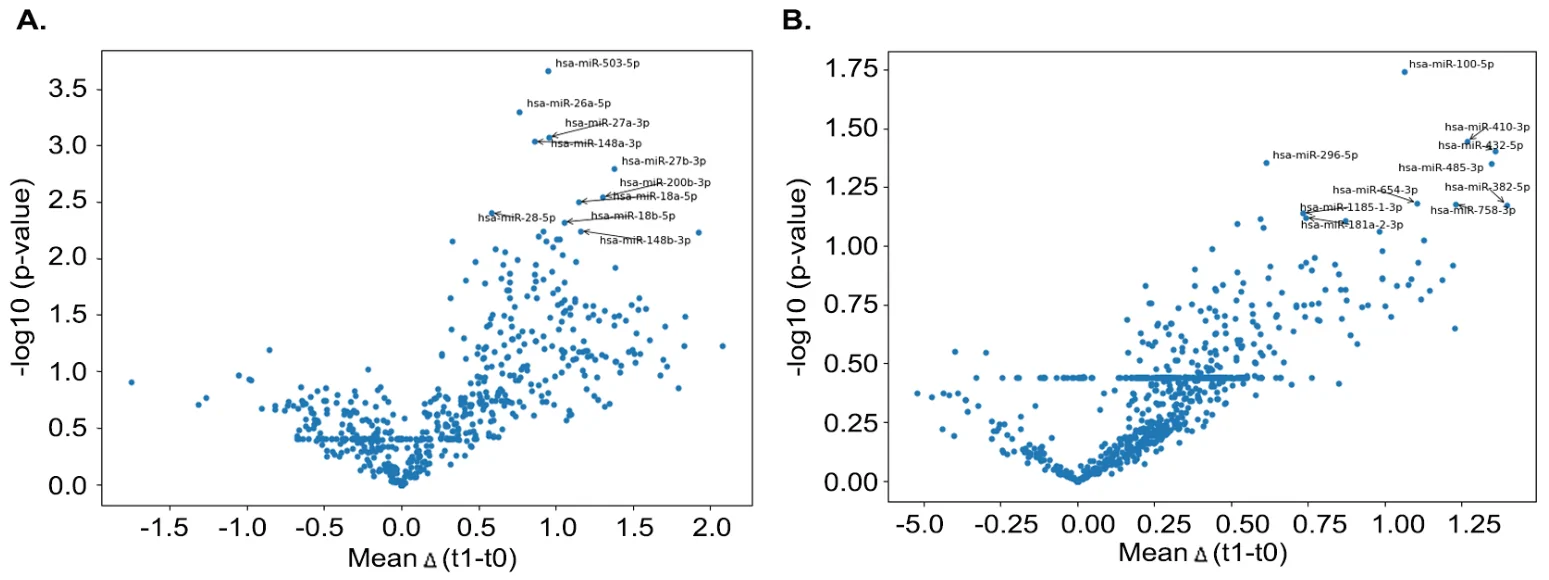
(**A**) Δ-miRNA volcano plot following cladribine treatment. (**B**) Δ-miRNA volcano plot following ocrelizumab (OCRE) treatment. Volcano plots show within-subject miRNA expression changes following cladribine or ocrelizumab treatment. All data points correspond to individual miRNAs, displayed by mean Δ-expression (t1 − t0) versus −log10(*p*-value). Representative miRNAs exhibiting the strongest treatment-associated changes are labelled. Fewer miRNAs exhibited large effect sizes, and the overall spread of Δ-expression values was narrower following cladribine treatment. Nevertheless, the volcano plot demonstrated a directional trend, indicating selective miRNA modulation following therapy. In contrast, the Δ-miRNA profile following ocrelizumab treatment showed a more modest pattern of change.

Volcano plot analysis of Δ-miRNA values following cladribine treatment revealed a pronounced and structured pattern of miRNA changes (Figure 2A). The volcano plot for cladribine (Figure 2A) shows strong changes in many miRNAs after treatment. Several miRNAs, including *hsa-miR-503-5p*, *hsa-miR-26a-5p*, *hsa-miR-27a-3p*, *hsa-miR-148a-3p*, and *hsa-miR-27b-3p*, show high increases in expression with strong significance. Most of the important changes are in the positive direction, while fewer miRNAs show decreases. Overall, this indicates that cladribine causes a broad and strong miRNA response. Large positive Δ-expression values with significant statistical support were shown by a group of miRNAs [17] which clearly separated from the background distribution that was centered around zero. The overall distribution indicated a coherent treatment-associated shift rather than random variability. The volcano plot for ocrelizumab (Figure 2B) shows that most miRNAs do not change strongly after OCR treatment, as they are clustered near the center with low significance. However, a few miRNAs, such as *hsa-miR-100-5p*, *hsa-miR-410-3p*, and *hsa-miR-432-5p*, show moderate increases in expression along with relatively higher significance. There are very few miRNAs showing strong decreases, indicating that the changes are mainly in one direction (increase). Overall, this suggests that ocrelizumab leads to selective and mild changes in specific miRNAs, rather than a broad or strong response across many miRNAs.

### 2.3. Top Treatment-Associated Δ-miRNAs

A concise summary of the representative miRNAs showing the strongest longitudinal changes and statistical support for each treatment group is provided in Table 2. This table presents representative miRNAs with the most significant Δ-expression values and the strongest statistical validation within each group, offering a concise summary of treatment-related signals. Together with the volcano plots, these findings demonstrate distinct molecular response patterns between cladribine and ocrelizumab at the miRNA level, similar to conclusions from the initial paper.

### 2.4. Ranking of Δ-miRNAs Following Cladribine and Ocrelizumab Treatment

**Figure 3:**
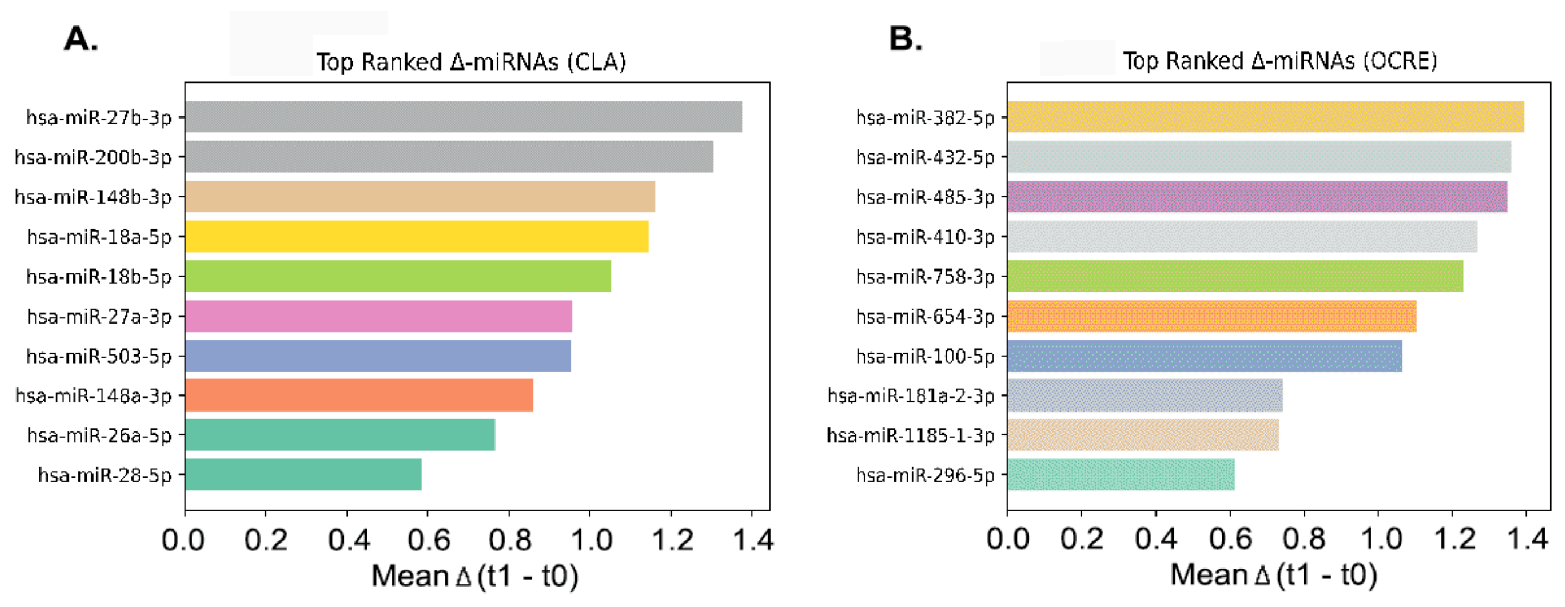
(**A**) Top-ranked Δ-miRNAs following cladribine treatment, ordered by mean within-subject Δ-expression (t1 − t0) across paired patients. (**B**) Top-ranked Δ-miRNAs following ocrelizumab (OCRE) treatment, ordered by mean Δ-expression (t1 − t0). Bars represent the magnitude of mean within-subject change for each miRNA.

Ranking of Δ-miRNAs following cladribine treatment, as previously conducted, we identified a subset of miRNAs exhibiting the largest and most consistent expression changes across patients [17] (Figure 3A). These miRNAs showed high mean Δ-expression values, reinforcing the structured response pattern observed in the volcano analysis [22]. Several of the top-ranked miRNAs belonged to related regulatory families, suggesting coordinated transcriptional modulation rather than isolated effects. A number of the cladribine-associated miRNAs are members of similar regulatory families, such as immune-regulatory miRNA clusters and the miR-27 family, indicating coordinated control of immune signaling pathways. The biological plausibility of the noted longitudinal alterations is strengthened by such family-level trends.

In contrast, ranking of Δ-miRNAs following ocrelizumab [23,24] treatment revealed a more selective response profile (Figure 3B). The top-ranked miRNAs demonstrated moderate but consistent Δ-expression changes, reflecting a subtler molecular response compared with cladribine [17]. Despite the reduced magnitude, the ranked miRNAs highlight specific treatment-associated signals that are also unique to the specific treatment, and no top miRNAs were found to overlap.

### 2.5. Stability of Δ-miRNAs Following Cladribine and Ocrelizumab Treatment

**Figure 4:**
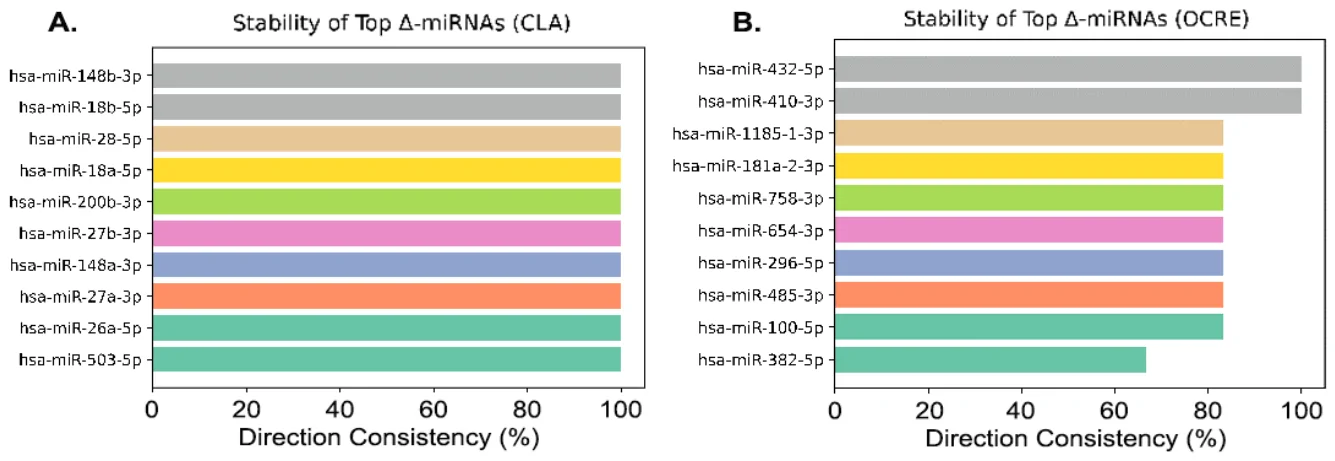
(**A**) Directional consistency of top-ranked Δ-miRNAs following cladribine treatment, expressed as the percentage of patients exhibiting Δ-expression in the same direction as the group mean. (**B**) Directional consistency of top-ranked Δ-miRNAs following ocrelizumab (OCRE) treatment. Bars indicate the proportion of subjects showing concordant directionality for each miRNA.

Stability analysis revealed a high degree of directional consistency among the top-ranked Δ-miRNAs following cladribine treatment (Figure 4A). Most miRNAs demonstrated consistent directionality across all or nearly all patients, indicating that observed Δ-expression changes were not driven by single individuals. This pattern indicates high directional consistency among cladribine-associated Δ-miRNAs across patients.

On the other hand, stability patterns of Δ-miRNAs linked to ocrelizumab therapy were more variable (Figure 4B). More inter-individual heterogeneity was reflected by the moderate consistency of certain miRNAs across patients, whereas others exhibited high directional consistency. This trend is consistent with ocrelizumab’s more focused and specific molecular actions on B cells compared to cladribine, which leads to a broader depletion of CD4+ and CD8+ T cell populations [25,26]. Compact and treatment-specific miRNA signatures for cladribine and ocrelizumab (OCRE) were obtained by integrating statistical evidence, ranking, and stability measures. These miRNAs are the most repeatable longitudinal signals found throughout the analytical process [27].

Table 3 shows that miRNAs were retained as robust treatment-associated signatures following integration of longitudinal change (Δ = t1 − t0), effect-size ranking, and directional stability across patients. Mean Δ-expression and directional stability percentages are shown separately for cladribine and ocrelizumab (OCRE) treatment groups.

### 2.6. Functional Consequences of miRNA Changes

We noted unique miRNAs not identified by [17] following either ocrelizumab or cladribine therapy. Many are related to MS, and a few are known to be elevated in progressive forms of the disease, as depicted in Table 4.

## 3. Discussion

Comprehensive quality control analyses confirmed that the miRNA expression dataset exhibits high technical integrity and is suitable for downstream longitudinal and treatment-specific analyses. The lack of extreme outliers in unsupervised principal component analysis and density (Figure 1A,B) and the strong overlap seen in sample-wise density distributions show that global expression patterns are very consistent across samples and are not dominated by batch-driven effects or technical artifacts. The observed variability appears to be mostly due to biological heterogeneity rather than noise, as this consistency is consistent with the Agilent microarray workflow’s efficient platform-level preprocessing and normalization [53]. Importantly, the QC analyses’ absence of noticeable non-biological factor separation or clustering lends credence to the reliability of later within-subject comparisons and treatment-stratified analyses, although this behavior is more indicative of technological resilience than biological signal loss. When taken as a whole, these QC results give assurance that the dataset provides a stable and trustworthy basis for examining long-term changes in miRNA expression linked to disease-modifying treatments, supporting the move to paired analyses that concentrate on treatment-induced molecular dynamics.

### 3.1. Longitudinal Pairing and Sample Structure

The rigorous criterion for full t1 − t0 pairing improves statistical robustness by allowing direct within-subject comparisons, decreasing confounding from inter-individual variability, even when the number of matched patients is limited. Internal validation of measurement consistency across timepoints and further support for data dependability are provided by the existence of several technical replicates for various patients [54]. When taken as a whole, the paired sample structure shown in Table 1 provides a solid basis for further studies that concentrate on changes in treatment-associated miRNA expression and supports the use of longitudinal difference-based methods.

The Δ-miRNA study demonstrated unique and treatment-specific molecular response patterns subsequent to cladribine and ocrelizumab therapy. Figure 2A demonstrates that cladribine therapy resulted in a coordinated alteration in miRNA expression, with several miRNAs showing significant positive Δ-expression and uniform directionality among patients. The inclusion of many miRNAs from related families among the most significant signals in Table 2 indicates organized regulatory alterations rather than random or isolated effects, aligning with the extensive immunomodulatory and immunological reconstitution processes associated with cladribine.

Conversely, the Δ-miRNA profile after ocrelizumab therapy exhibited a more subdued pattern of alteration (Figure 2B), with a reduced number of miRNAs demonstrating significant changes in expression. This selective response likely indicates the specific mechanism of B-cell depletion and the anticipation of more nuanced downstream transcriptional regulation, as previously shown to be the case for B cell differentiation [55]. Although the quantity of change is diminished, the discernible directionality and consistency among the predominant miRNAs suggest that ocrelizumab medication is still linked to certain miRNA-level modifications.

Collectively, our data illustrate that cladribine and ocrelizumab elicit unique miRNA response signatures, underscoring therapy-specific molecular reconfiguration rather than a common therapeutic impact. The miRNAs listed in Table 2 constitute a targeted selection of candidates for subsequent integrative studies and endorse the application of Δ-based, within-subject methodologies to elucidate treatment-related molecular dynamics. These results establish a molecular framework for subsequent stability-aware and machine-learning-based analyses aimed at characterizing treatment response patterns. The miRNAs found in this research most likely represent immune remodeling mechanisms linked to therapy that take place in response to multiple sclerosis disease-modifying therapies. A number of the cladribine-associated miRNAs have previously been connected to inflammatory signaling pathways and immunological control. For instance, miR-503-5p has been linked to the control of inflammatory signaling networks and immune cell proliferation, indicating that its elevated expression may represent immunological reconstitution activities after cladribine-induced lymphocyte depletion. Similar to this, miR-26a-5p has been shown to control cytokine signaling pathways and T-cell activation, indicating that it may play a part in influencing adaptive immunological responses during treatment-associated immune rebalancing. The coordinated overexpression of members of the miR-27 family, such as miR-27a-3p and miR-27b-3p, which are recognized regulators of inflammatory responses and macrophage polarization, may suggest more extensive modulation of innate immune signaling after cladribine treatment. The miRNA profile seen after ocrelizumab therapy, on the other hand, is more selective and contains miR-100-5p, a regulator that was previously linked to immune regulatory pathways and B-cell development. This finding is in line with ocrelizumab’s mode of action, which modifies downstream immune signaling networks by specifically eliminating CD20-positive B cells. When combined, these findings imply that the miRNA signatures found in this study may represent therapy-specific transcriptional regulatory programs that reflect different immune modulation mechanisms brought on by ocrelizumab-mediated B-cell depletion and cladribine-mediated immune reconstitution. These miRNAs are physiologically reasonable candidates for further research aiming at comprehending molecular responses to disease-modifying medications in multiple sclerosis, even though functional validation will be necessary.

The ranking analysis refined the Δ-miRNA signals identified in earlier analyses into treatment-specific molecular signatures. Data in Figure 3A illustrates that cladribine therapy resulted in a significant and coordinated alteration in the expression of a specific group of miRNAs, along with extensive immunomodulatory and immune-reconstitution effects. The recurrence of miRNAs from the highest-ranked candidates in Table 2 indicates the existence of organized regulatory responses rather than random fluctuation [56].

Conversely, ocrelizumab therapy yielded a more subdued ranking profile (Figure 3B), marked by a reduced number of miRNAs exhibiting moderate effect sizes. This pattern corresponds with the intended mode of action of ocrelizumab and indicates selective downstream transcriptional regulation. The found ranking miRNAs for ocrelizumab offer insight into certain biological pathways activated post-therapy [57]. The graded Δ-miRNA profiles indicate that cladribine and ocrelizumab elicit unique and treatment-specific miRNA response patterns.

### 3.2. Interpretation of Stability Findings

The stability analysis presented provides critical validation of the treatment-associated Δ-miRNA signals identified in earlier results. The idea that these variations represent a common molecular response rather than individual-specific fluctuations is supported by the high directional consistency of cladribine-associated miRNAs across patients, as illustrated in (Figure 4A). Broad immunomodulatory and immune reconstitution effects in both the periphery and CSF are linked to cladribine treatment and align with its stability profile [58,59]. The ocrelizumab-associated miRNA profile, on the other hand, shows significant inter-individual variability (Figure 4B), indicating a more varied molecular response. This result suggests that downstream transcriptional effects may differ across individuals and is consistent with the targeted mechanism of B-cell depletion. Crucially, despite their decreased overall stability, a number of miRNAs continue to show strong directional consistency, confirming their significance as treatment-associated signals. The final treatment-associated Δ-miRNA signatures identified in this study represent the most reproducible longitudinal molecular alterations observed across the analytical workflow. These signatures capture miRNAs that consistently respond to therapy instead of showing random variation or isolated statistical significance [60]. They do this by combining longitudinal change, effect magnitude, and directional stability across individuals. The cladribine-associated signature is defined by precisely coordinated miRNA regulation exhibiting consistently high directional stability among patients, signifying a collective molecular response to treatment. This pattern aligns with the extensive immunomodulatory and immune-reconstitution effects of cladribine, reinforcing the notion that these miRNAs represent authentic treatment-related regulatory mechanisms.

In contrast, the signature associated with ocrelizumab shows stable but more varied longitudinal modulation, which has also been noted in human T cells in response following ocrelizumab treatment; although ocrelizumab-associated Δ-miRNAs exhibit greater inter-individual variability than cladribine-associated signals, several miRNAs still demonstrate consistent directional changes across patients. This suggests that the therapy induces selective but reproducible transcriptional modulation, rather than the broader coordinated response observed following cladribine treatment. These results show that strong changes in miRNA levels that are linked to treatment can be found without relying only on isolated statistical significance. By focusing on longitudinal consistency and reproducibility, we developed a biologically based way to understand therapy-specific molecular responses and a clear list of candidate miRNAs for future validation studies.

## 4. Materials and Methods

### 4.1. Dataset

The publicly accessible Gene Expression Omnibus (GEO), with accession number GSE230064, provided the miRNA expression dataset utilized in this investigation. PBMC samples from patients with relapsing-remitting multiple sclerosis (RRMS) receiving high-efficacy disease-modifying treatments, such as ocrelizumab (OCRE; *n* = 14) and cladribine (CLA; *n* = 11), as well as untreated control individuals (CTRL; *n* = 15), are included in the collection. While control participants were sampled just once, samples from treated patients were taken at baseline (t0) and six months after treatment started (t1), as mentioned in Table 5. The original trial reported that ocrelizumab was given as 300 mg intravenous infusions twice over a two-week period, whereas cladribine pills were given at a cumulative dosage of 1.75 mg/kg over five days. The collection includes 65 microarray samples and 40 distinct participants overall, which reflects the inclusion of technical duplicates and repeated measurements throughout time. Only treated patients with full paired t1 − t0 samples were kept for the current longitudinal study after technical replicates were collapsed, resulting in a final cohort of 10 paired patients (4 CLA, 6 OCRE).

### 4.2. Study Cohort and Experimental Design

The dataset comprises peripheral blood mononuclear cell (PBMC) samples collected from individuals with relapsing-remitting MS (RRMS) treated with high-efficacy disease-modifying therapies, as well as untreated control subjects. RRMS patients received either cladribine (CLA; *n* = 11 patients) or ocrelizumab (OCRE; *n* = 14 patients). For treated patients, blood samples were collected at baseline prior to treatment initiation (t0) and again at 6 months post-treatment (t1). Untreated control subjects (*n* = 15) were sampled once at baseline. In total, 65 samples were included in the analysis. miRNA expression profiling was performed using an Agilent non-coding RNA microarray platform. Sample-level metadata, including treatment group, timepoint, tissue source, and cell type, were extracted directly from the GEO series matrix file. Although the GEO cohort included 11 cladribine-treated and 14 ocrelizumab-treated patients, only individuals with complete baseline (t0) and post-treatment (t1) samples were eligible for longitudinal Δ-miRNA analyses. After patient-level pairing and consolidation of technical replicates, the final paired cohort comprised 4 cladribine-treated and 6 ocrelizumab-treated patients. The statistical data were interpreted with caution due to the small number of matched patients that were included in each therapy arm within this study. Rather than strictly testing the hypothesis, the major objective of statistical testing was to prioritize the signals, and as a result, statistical significance was interpreted in conjunction with effect magnitude and stability criteria.

A detailed summary of sample characteristics and study design is provided in Table 6.

### 4.3. Quality Control and Preprocessing of miRNA Expression Data

Quality control (QC) analyses were performed to assess the global structure, distributional consistency, and technical integrity of miRNA expression data prior to downstream statistical analyses. miRNA expression values were extracted from the GEO series matrix file associated with accession GSE230064 and organized into a sample-by-feature expression matrix. No additional filtering, re-normalization, or batch correction was applied beyond the preprocessing performed by the original platform pipeline.

To make it easier to understand and keep the variance stable across the range of measured intensities, expression values were log2-transformed using log2(x + 1) [61]. We used sample-wise density plots to look at the global expression distributions across samples. This helped us make sure that the expression profiles were consistent and find any technical outliers. We conducted unsupervised principal component analysis (PCA) on the log2-transformed expression matrix to find the main sources of variance and any possible sample-level artifacts or batch-driven structure. PCA was performed without the inclusion of phenotypic or treatment labels, guaranteeing an impartial evaluation of data quality [62].

### 4.4. Identification of Paired Samples and Handling of Technical Replicates

To enable longitudinal within-subject analyses, patient-level pairing of samples collected at baseline (t0) and 6 months post-treatment (t1) was performed for cladribine- and ocrelizumab-treated individuals. We obtained sample metadata from the GEO series matrix file. This included the treatment assignment, timepoint, and unique sample identifiers [63]. A consistent patient-level identification was created by taking common prefixes from sample titles and using them to group together several technical measures that belonged to the same person. For every patient who received therapy, samples were organized by treatment arm and patient identifier. To be included in paired analyses, there had to be at least one baseline (t0) sample and one post-treatment (t1) sample. When there was more than one technical replicate for a patient and timepoint, the number of replicates was recorded to check for technical redundancy. All patients involved in subsequent analyses possessed complete t1 − t0 pairings, and none were omitted due to absent timepoints.

### 4.5. Technical Replicates and Computation of Longitudinal miRNA Changes

To measure changes in miRNA expression over time in the same patient after therapy, technical replicates that were taken at the same time and for the same patient were combined before the statistical analysis [53]. We obtained the miRNA expression values from the processed expression matrix and changed them to log2(x + 1) to make the variance more stable and lower the effect of very high or low intensity values.

For each patient i, miRNA j, and timepoint t (baseline t0 or post-treatment t1), we combined many technical replicates by finding their arithmetic mean [53]. Formally, the collapsed expression value was defined as follows:

$$\bar{E_{i,j,t}}=\frac{1}{n_{i,t}}\sum_{k=1}^{n_{i,t}}\;\log_{2}\left(E_{i,j,t,k}+1\right),$$

where Ei,j,t,kE_{i,j,t,k}Ei,j,t,k denotes the raw expression value of miRNA *j* for the *k*-th technical replicate of patient *i* at timepoint *t*, and ni,tn_{i,t}ni,t represents the number of available technical replicates for that patient and timepoint.

Longitudinal miRNA expression changes were then computed on a per-patient basis as the difference between post-treatment and baseline expression levels [62]:

$$\Delta_{\left\{i,j\right\}}=\;{\bar{E}}_{\left\{i,j,t^{1}\right\}}-\;{\bar{E}}_{\left\{i,j,t^{0}\right\}},$$

where Δi,jΔ_{i,j}Δi,j represents the within-subject change in expression of miRNA *j* for patient *i*. Positive Δ values indicate increased expression following treatment, whereas negative Δ values indicate decreased expression [64]. Δ-miRNA matrices were computed separately for cladribine-treated and ocrelizumab-treated patients and retained in comma-separated value (CSV) format for downstream statistical testing and visualization.

To identify treatment-associated miRNA changes, we performed statistical testing on within-subject Δ-miRNA values derived from paired baseline (t0) and post-treatment (t1) samples. For each patient and each miRNA, Δ-expression was computed as the difference between post-treatment and baseline expression levels (Δ = t1 − t0), following collapse of technical replicates by the mean on a log_2_(x + 1) scale.

Separate analyses were performed for each treatment arm, cladribine and ocrelizumab, to prevent confounding molecular effects specific to each drug. A one-sample *t*-test was conducted for each miRNA within each treatment group to see if the mean Δ-expression substantially deviated from zero, in line with the null hypothesis of no systematic treatment-related change.

For each miRNA, the following statistics were computed: mean Δ-expression, standard deviation of Δ, t-statistic, and nominal *p*-value. Multiple-testing correction was performed using the Benjamini–Hochberg false discovery rate (FDR) procedure across all tested miRNAs within each treatment arm [65]. Due to the limited sample size and the presence of miRNAs with insufficient within-subject variance, FDR estimates were conservatively interpreted and reported primarily for transparency. All statistical analyses were implemented in Python using SciPy and stats models libraries. (Python: 3.12.13, SciPy: 1.16.3, pandas: 2.2.2, NumPy: 2.0.2).

### 4.6. Stability and Consistency Analysis of Treatment-Associated Δ-miRNAs

To assess the robustness of treatment-associated Δ-miRNAs, a stability and consistency analysis was performed using subject-level Δ-miRNA matrices. Analyses were conducted separately for cladribine and ocrelizumab treatment arms.

We measured directional consistency for each miRNA by finding the percentage of patients whose Δ-expression was in the same direction as the group-level mean Δ. This statistic gives an easy-to-understand way to tell if changes seen in therapy are common to all participants or only to a few [60]. We also performed a leave-one-out (LOO) sensitivity analysis by taking away one individual at a time and calculating the mean Δ-expression again [64]. We looked at just how sensitive group-level estimations are to individual participants by looking at how different LOO means were. Before using biological interpretation, these extra measures were utilized to check the stability of the top-ranked Δ-miRNAs.

We used a multi-evidence framework to combine the data to come up with strong treatment-related miRNA signatures. For each treatment arm (cladribine and ocrelizumab), miRNAs were maintained only if they exhibited (i) consistent longitudinal change (Δ = t1 − t0), (ii) high ranking based on impact size, and (iii) substantial directional stability across individuals. This integrated method puts more value on consistency and repeatability than on standalone statistical significance. It produces treatment-specific miRNA signatures that show sustained longitudinal molecular responses.

## 5. Limitations

This study possesses certain limitations that must be acknowledged when evaluating the results. First, the initial dataset (GSE230064) had untreated control participants sampled just once, in contrast to the treated groups, samples from which were collected at baseline and six months post-therapy. Consequently, we were unable to implement the identical longitudinal, within-subject methodology for controls or assess whether treatment-related microRNA profiles reverted to a healthy baseline. Second, the publicly accessible metadata includes fundamental demographic and treatment information but lacks Expanded Disability Status Scale (EDSS) ratings. As a result, we could not analyze the associations between miRNA expression and disability development, hence constraining the clinical interpretability of our results. Third, after the consolidation of technical duplicates and the necessity for matched baseline and follow-up samples, only four patients treated with cladribine and six patients treated with ocrelizumab were available for longitudinal analysis. The limited sample size diminishes statistical power and may restrict the generalizability of the findings. Fourth, functional validation of the identified miRNA candidates was beyond the scope of this study. Ultimately, given that this was a single-cohort exploratory investigation, subsequent research should involve larger, well-characterized cohorts and longitudinal sampling of healthy individuals to corroborate these findings and determine if miRNA alterations signify a reversion to physiological baselines.

## 6. Conclusions

This study conducted a longitudinal examination of treatment-related miRNA dynamics in MS by within-subject Δ-miRNA profiling. Through the integration of statistical testing, effect-size ranking, and stability evaluation across people, we discerned consistent and treatment-specific miRNA response patterns subsequent to cladribine and ocrelizumab medication.

The findings indicate that cladribine is linked to a highly coordinated miRNA response with significant directional consistency among patients, while ocrelizumab elicits a more selective and variable miRNA modulation pattern. The definitive miRNA signatures presented in Table 2 underscore molecular alterations that are consistent among people and unlikely to be influenced by individual-specific influences. This work prioritizes repeatability and longitudinal consistency above isolated statistical significance as an essential criterion for identifying physiologically relevant treatment-associated miRNA signals. These findings collectively offer a consistent molecular framework for comprehending therapy-specific immune modulation in MS and build a solid platform for future translational and mechanistic research.

Multiple avenues can enhance and reinforce the conclusions of this research. Initially, the validation of the discovered miRNA signatures in larger, independent cohorts is crucial to ascertain their generalizability across varied patient groups and therapeutic contexts. The inclusion of extended follow-up periods may elucidate the temporal stability of these miRNA responses and their correlation with prolonged therapy benefits. Secondly, the integration of the discovered miRNAs with downstream mRNA, proteomic, or immune-cell-specific data may clarify the molecular pathways by which these miRNAs influence treatment response. Multi-omics methodologies might enhance the biological understanding of therapy-specific regulation networks. Subsequently, future research may investigate the therapeutic significance of these miRNA signatures by analyzing correlations with therapy efficacy, illness progression, or long-term results. With proper validation, persistent longitudinal miRNA signatures may function as relevant biological markers to assist in treatment monitoring and tailored therapeutic approaches in MS. This study has several limitations. First, the number of patients with complete longitudinal pairs was small (*n* = 4 for cladribine and *n* = 6 for ocrelizumab), which limits statistical power. Second, the analysis relied on publicly available microarray data without access to detailed clinical outcomes, preventing correlation of miRNA changes with treatment response or disease activity. Third, only a single post-treatment timepoint (6 months) was available, limiting evaluation of temporal dynamics. Finally, functional validation of the identified miRNA candidates was beyond the scope of this study.

## Figures and Tables

**Table 1:** Summary of paired samples included in longitudinal analyses.

Treatment	Number of Patients	Complete t1 − t0 Pairs	Patients with Technical Replicates
Cladribine	4	4	4
Ocrelizumab	6	6	6

This paired cohort formed the basis for subsequent longitudinal analyses assessing within-subject miRNA expression changes following treatment.

**Table 2:** Selected Δ-miRNAs associated with cladribine and ocrelizumab treatment: summary of representative miRNAs showing treatment-associated changes following cladribine (CLA) and ocrelizumab (OCRE) therapy. Mean Δ-expression (t1 − t0), (SD)standard deviation [18], test statistic, and nominal *p*-values are reported for each miRNA; false discovery rate (FDR) values are provided for transparency but interpreted conservatively due to limited sample size.

Treatment	miRNA	Mean Δ (t1 − t0)	SD Δ	t-Statistic	*p*-Value	FDR (q-Value)
CLA	hsa-miR-503-5p	0.952332	0.088553	21.508778	2.20 × 10^−4^	0.134808
CLA	hsa-miR-26a-5p	0.765690	0.093851	16.317194	5.01 × 10^−4^	0.141283
CLA	hsa-miR-27a-3p	0.955557	0.139707	13.679444	8.45 × 10^−4^	0.141283
CLA	hsa-miR-148a-3p	0.861446	0.129696	13.284068	9.22 × 10^−4^	0.141283
CLA	hsa-miR-27b-3p	1.376618	0.250515	10.990301	1.61 × 10^−3^	0.197760
OCRE	hsa-miR-100-5p	1.063450	0.754590	3.452085	1.82 × 10^−2^	0.752265
OCRE	hsa-miR-410-3p	1.267997	1.090543	2.848071	3.59 × 10^−2^	0.752265
OCRE	hsa-miR-432-5p	1.359277	1.201364	2.771462	3.93 × 10^−2^	0.752265
OCRE	hsa-miR-296-5p	0.612689	0.562015	2.670351	4.43 × 10^−2^	0.752265
OCRE	hsa-miR-485-3p	1.347491	1.239347	2.663228	4.47 × 10^−2^	0.752265

**Table 3:** Final robust treatment-associated Δ-miRNA signatures derived from integrated longitudinal analysis.

Treatment	miRNA	Mean Δ (t1 − t0)	Directional Stability
CLA	hsa-miR-503-5p	+0.95	100
CLA	hsa-miR-26a-5p	+0.77	100
CLA	hsa-miR-27a-3p	+0.96	100
CLA	hsa-miR-148a-3p	+0.86	100
CLA	hsa-miR-27b-3p	+1.38	100
OCRE	hsa-miR-100-5p	+1.06	83
OCRE	hsa-miR-410-3p	+1.27	83
OCRE	hsa-miR-432-5p	+1.36	83
OCRE	hsa-miR-296-5p	+0.61	83
OCRE	hsa-miR-485-3p	+1.35	83

**Table 4:** Overview of potential cellular targets: Summary of potential miRNA cellular targets unique to either OCRE or CLA. Only hsa-miR-27b-3p was common between the prior analysis by Arisi et al. and our current t0- and t1-paired analysis.

Treatment	miRNA	Validated/Predicted Targets	Function in Relation to MS	Citations
CLA	hsa-miR-503-5p	CDC25a, CCND1, BCL2	Not directly implicated in MS; regulation of cell cycle and apoptosis under stress	[28,29]
CLA	hsa-miR-26a-5p	SLC1A1 Glutamate receptor signaling; inversely correlated to DLG4	Upregulated in Natalizumab and IFNβ treatment	[30,31,32]
CLA	hsa-miR-27a-3p	PPARγ, Wnt3a, Wnt-β-catenin signaling pathway modulator	Th17/Treg balance, OPC differentiation; linked to RRMS → SPMS transition	[33,34,35]
CLA	hsa-miR-148a-3p	HLA-G, DNMT1	Epigenetic regulation of immune tolerance; low serum levels linked to primary progressive MS	[36,37]
CLA	hsa-miR-27b-3p	PPARγ, FOXO1	Impacts lipid metabolism and Th1/Th2 balance	[33,38,39]
OCRE	hsa-miR-100-5p	mTOR, IGF1R	Not directly implicated in MS; modulates autophagy and cell survival	[40,41]
OCRE	hsa-miR-410-3p	STAT3, ELAVL4	Reduced in RRMS; impairs pro-inflammatory cytokine secretion	[42,43,44]
OCRE	hsa-miR-432-5p	ADAR1, Syt7	Elevated in gray matter lesions and in progressive MS patients; regulation of RNA editing and innate immune sensing	[45,46,47]
OCRE	hsa-miR-296-5p	IKBKE, NumbL, STAT5a	Regulated by circular RNA linked to MS; modulates NF-κB signaling	[48,49]
OCRE	hsa-miR-485-3p	PGC-1α, STAT3	Downregulated in RRMS compared to control patients; regulates Th17 response in experimental autoimmune encephalitis, mitochondrial biogenesis, and energy homeostasis	[50,51,52]

**Table 5:** Summary of dataset composition and sample selection from GEO dataset GSE230064.

Category	Number
Total subjects	40
Cladribine-treated patients	11
Ocrelizumab-treated patients	14
Untreated control subjects	15
Total microarray samples (including technical replicates)	65
Baseline samples (t0)	33
Follow-up samples (t1)	32
Patients with complete paired samples used in longitudinal analysis	10
Cladribine paired patients	4
Ocrelizumab paired patients	6

**Table 6:** Summary of study design and sample characteristics for cladribine- and ocrelizumab-treated patients included in the longitudinal Δ-miRNA analysis. The table reports sample counts, timepoints, and paired patient structure prior to consolidation of technical replicates.

Characteristic	Cladribine	Ocrelizumab	Total
Total samples (including technical replicates of t0 and t1)	29	36	65
Baseline samples (t0)	15	18	33
Post-treatment samples (t1)	14	18	32
Patients with complete paired t1 − t0 samples	4	6	10
Samples per patient (range)	1–3	1–3	—
Presence of technical replicates	Yes	Yes	Yes
Tissue source	Peripheral blood	Peripheral blood	—
Cell type	PBMC	PBMC	—
Expression platform	GEO microarray	GEO microarray	—

## Data Availability

The miRNA expression data analyzed in this study were obtained from the NCBI GEO # accession number GSE230064, a publicly accessible repository hosting high-throughput functional genomics datasets. The dataset comprised longitudinal miRNA expression profiles from MS patients treated with cladribine or ocrelizumab, including paired baseline and post-treatment samples. The data were downloaded from GEO, curated, and reanalyzed locally for the purposes of this study.
